# Chimeric antigen receptor‐T cell therapies: The changing landscape

**DOI:** 10.1002/jha2.340

**Published:** 2021-11-29

**Authors:** Salyka M. Sengsayadeth, Bhagirathbhai R. Dholaria, Bipin N. Savani, Olalekan O. Oluwole

**Affiliations:** ^1^ Section of Hematology and Stem Cell Transplant Vanderbilt Ingram Cancer Center Department of Medicine Vanderbilt University Medical Center Nashville Tennessee USA; ^2^ Veterans Affairs Medical Center Tennessee Valley Healthcare System Nashville Tennessee USA

**Keywords:** chimeric antigen receptor, leukemia, multiple myeloma, non‐Hodgkin lymphoma, T cell

Since the advent of the first Food and Drug Administration (FDA)‐approved chimeric antigen receptor (CAR‐T) cell therapy in 2017 for aggressive large B cell lymphoma, the excitement around this nascent, cutting‐edge therapy has only continued to grow in the arena of Hematology and Oncology. CAR‐T cell therapy involves harnessing a patient's own or donor immune cells and subsequently engineering these T cells to recognize specific antigens on tumor cells and in turn causing cancer cell death. The development of this new treatment modality has been a beacon of the direction of where cancer treatment is headed in the future. Leaving behind the diffuse cytotoxic side effects of standard chemotherapy in its path, CAR‐T cell therapy has become an effective and well‐tolerated therapeutic option for many patients with relapsed or refractory hematologic malignancies. However, as experience with CAR‐T cell therapy continues to develop and data regarding long‐term efficacy and side effects mature, we are learning that there is more to learn. Herein this special issue, we discuss what we know, what the current challenges are, and what we need to learn in the future regarding this avant‐garde therapy that has changed the landscape for so many with life‐threatening hematologic malignancies (Table 1).

**TABLE 1:** Summary of articles

**Authors**	**Title**	**Key highlights**
Sengsayadeth et al.[1]	Overview of approved CAR‐T therapies, ongoing clinical trials, and its impact on clinical practice	Reviewed currently approved CAR‐T therapies. Key clinical trials with the potential to impact clinical practice Changes in treatment paradigm with the addition of CAR‐T therapies
Iqbal et al.[2]	New Indications and platforms for CAR‐T therapy in lymphomas beyond DLBCL	Currently approved CAR‐T therapies for FL and MCL Next‐generation of CAR‐T therapies in lymphoma under investigation Allogenic CAR‐T, novel CAR‐T constructs, and future directions
Patel et al.[4]	CAR‐T cell therapy in solid tumors: a review of current clinical trials	Current and upcoming trials in solid organ malignancies Challenges with developing safe and effective CAR‐T therapy for solid organ malignancy Strategies to overcome limitations
Taneja et al.[7]	CAR‐T‐OPENIA: chimeric Antigen Receptor T‐cell therapy Associated Cytopenias	Prevalence and severity of cytopenia after CAR‐T therapy Possible pathophysiology of early and late cytopenia after CAR‐T therapy Framework for clinical monitoring and management
Bhaskar et al.[5]	Role of bridging therapy during chimeric antigen receptor T cell therapy	Role of bridging therapy in improving safety and efficacy to CAR‐T therapy Published experience with bridging therapy Choice and administration of bridging therapy for NHL, B ALL, and MM
Jain et al.[3]	Imagining the cell therapist: future CAR T cell monitoring and intervention strategies to improve patient outcomes	Evolution of a clinical Cell Therapist Optimizing host‐ tumor‐immune interaction to improve the efficacy of CAR‐T therapy Real‐time in vivo monitoring of CAR‐T cell in tumor response Monitoring of anti‐CAR immunity and strategies to overcome CAR‐T cell rejection
Gatwood et al.[6]	Chimeric antigen receptor (CAR) T‐cell therapy: challenges and framework of outpatient administration	Current experience with outpatient CAR‐T therapy administration Potential benefits and limitations of outpatient CAR‐T administration Components of outpatient CAR‐T program and metrics of success

Abbreviations: B ALL, B cell acute lymphoblastic leukemia; DLBCL, diffuse large B cell lymphoma; FL, follicular lymphoma; MCL, mantle cell lymphoma; MM, multiple myeloma; NHL, non‐Hodgkin lymphoma.

First, we must know what has happened so far and where we are in today's day and age. Currently, five FDA‐approved CAR‐T cell products are approved for the treatment of patients with relapsed, refractory B cell lymphomas, mantle cell lymphoma, acute lymphoblastic leukemia, and multiple myeloma. In this special issue, our first two articles address what the current FDA‐approved CAR‐T products are, the ongoing CAR‐T clinical trials, and how these data are affecting our day‐to‐day clinical practice.[1, 2] We also discuss the new indications and platforms of therapy in lymphomas beyond diffuse large B cell lymphoma and how it is impacting the treatment for patients even in indolent forms of non‐Hodgkin lymphoma.[2] Importantly, we also look at where CAR‐T fits in the sequence of currently available treatment options; something increasingly important to discern given the multitude of choices available. As the experience with CAR‐T therapy matures, novel tools are needed for optimizing host‐tumor immune interaction, real‐time monitoring of in vivo CAR‐T cell activity and tumor responses. As this translational science moves into the clinical space, the next generation of cellular therapists will have many tools to improve the safety and efficacy of CAR‐T therapy as described by Jain et al.[3]

As with many therapies that are developed in the space of oncology such as molecularly targeted therapies, it is often the plan to expand their use to many other targets and different tumor types. This is no different with CAR‐T cell therapy, and of course, the natural area of expansion beyond hematologic malignancies is that of solid tumor malignancies. In this issue, Patel et al. discuss the ongoing trials of CAR‐T cell therapies in solid tumors, of which thereof a plethora, and where future directions may hold promise in the area of solid tumors.[4] It is very much in the realm of possibility that CAR‐T cell therapies will be approved for the first solid tumor type in the not‐so‐distant future.

As the expansion of CAR‐T cell therapy goes beyond hematologic malignancies, knowledge about optimizing treatment, pre‐, during, and post‐cellular therapy will continue to multiply. One important question that is often encountered during CAR‐T consideration is whether and how to reduce the tumor burden prior to cellular therapy. There are data that argue for and against bridging therapy as some of the pivotal trials allowed it while others did not. Bhaskar et al. reviewed the pros and cons of bridging therapy and how and when to consider this as part of the road to CAR‐T cell therapy.[5] As more and more centers become experienced in the implementation of CAR‐T, the safety and cost‐effectiveness of primary outpatient management of patients will continue to be an attractive option, given that most transplant and cellular therapy centers are already equipped with the staffing and logistics to make this a safe approach for patients as discussed by Gatwood et al.[6] Increasing knowledge of the optimal management of the unique and potentially severe complications such as cytokine release syndrome and immune effector cell‐associated neurotoxicity syndrome will continue to cultivate a more protocolized management strategy to minimize the risks to the patients, particularly as the age range of eligible patients continues to push the boundaries. Fortunately, cytokine release syndrome and immune effector cell‐associated neurotoxicity syndrome are well described and well managed at experienced centers, and this will only continue to expand. That said, we are also learning about the other important side effects of CAR‐T cell therapy such as cytopenias that do impact patients in terms of the need for transfusion, growth factor support, and infection risk. We are learning that these issues can last beyond even the initial transplant center care and require continued monitoring and collaboration with referring centers as discussed in CAR‐T‐OPENIA by Taneja et al.[7]

The excitement of CAR‐T cell therapy has translated to a continued burgeoning interest in expanding this therapy well beyond its current scope today. It is expected that the future of CAR‐T will encompass not only hematologic malignancies but solid tumors as well and perhaps even beyond the field of hematology/oncology. It is foreseeable that the time to treatment will significantly shorten as the infrastructure and access to CAR‐T treatment becomes more increasingly available. Challenges such as quick access and cost will continue to remain issues for patients and cost‐benefit discussions need to continue for those in need of this potentially life‐saving therapy. As data about long‐term survivors of CAR‐T cell therapy continues to mature, perhaps the application of the word cure may be conceivable for some patients. Ultimately, that is the hope that we can offer patients on the horizon of this changing landscape of CAR‐T cell therapy.

## References

[1] Sengsayadeth S , Savani B , Oluwole O , Dholaria B . Overview of approved CAR‐T therapies, ongoing clinical trials, and its impact on clinical practice. EJHaem. 2022;3(Suppl. 1):6‐10. 10.1002/jha2.338 PMC9175669 35844299

[2] Iqbal M , Savani B , M Hamadani . New Indications and platforms for CAR‐T therapy in lymphomas beyond DLBCL. EJHaem. 2022;3(Suppl. 1):11‐23. 34988550 10.1002/jha2.323 PMC8725814

[3] Jain MD , Spiegel JY . Imagining the cell therapist: future CAR T cell monitoring and intervention strategies to improve patient outcomes. EJHaem. 2022;3(Suppl. 1):46‐53. 10.1002/jha2.357 PMC9175904 35844298

[4] Patel U , Abernathy J , Sengsayadeth S , Savani B , Oluwole O , Dholaria B . CAR T cell therapy in solid tumors: a review of current clinical trials. EJHaem. 2022;3(Suppl. 1):24‐31. 10.1002/jha2.356 PMC9175685 35844304

[5] Bhaskar S , Dholaria B , Sengsayadeth S , Savani B , Oluwole O . Role of bridging therapy during chimeric antigen receptor T cell therapy. EJHaem. 2022;3(Suppl. 1):39‐45. 10.1002/jha2.335 PMC9175845 35844303

[6] Gatwood KS , dholaria B , Lucena M , Baer B , Savani B , Oluwole O . Chimeric antigen receptor (CAR) T‐cell therapy: challenges and framework of outpatient administration. EJHaem. 2022;3(Suppl. 1):54‐60. 10.1002/jha2.333 PMC9176074 35844300

[7] Taneja A , Jain T . CAR‐T‐OPENIA: chimeric antigen receptor T‐cell therapy associated cytopenias. EJHaem. 2022;3(Suppl. 1):32‐38. 10.1002/jha2.350 PMC9175816 35844301

